# Protein Expression Dynamics in Breast Cancer Cells Exposed to Nano-Encapsulated Tarin, the Taro Lectin

**DOI:** 10.3390/pharmaceutics18091123

**Published:** 2026-09-07

**Authors:** Raiane V. Cardoso, Patricia R. Pereira, Cyntia S. Freitas, Yuri P. Souza, Dário E. Kalume, Giovani Carlo Verissimo da Costa, Carlos A. Conte-Junior, Vania Margaret Flosi Paschoalin

**Affiliations:** 1 Departamento de Bioquímica, Instituto de Química, Universidade Federal do Rio de Janeiro, Av. Athos da Silveira Ramos 149, Sala 545, Cidade Universitária, Rio de Janeiro 21941-909, RJ, Brazil; raiane.gnb@hotmail.com (R.V.C.); patriciarp@iq.ufrj.br (P.R.P.); freitas.cs@pos.iq.ufrj.br (C.S.F.); conte@iq.ufrj.br (C.A.C.-J.); 2 Unidade de Espectrometria de Massas e Proteômica, Instituto de Bioquímica Médica Leopoldo de Meis, Universidade Federal do Rio de Janeiro, Av. Carlos Chagas Filho 373, Cidade Universitária, Rio de Janeiro 21941-599, RJ, Brazil; yuri.souza@bioqmed.ufrj.br; 3 Fundação Oswaldo Cruz, Instituto Oswaldo Cruz, Av. Brasil 4365, Manguinhos, Rio de Janeiro 21040-360, RJ, Brazil; kalume@ioc.fiocruz.br; 4 Instituto de Saúde, Universidade Federal Fluminense, Rua Dr. Silvio Henrique Braune 22, Centro, Nova Friburgo 28625-650, RJ, Brazil; giovaniverissimo@id.uff.br

**Keywords:** MDA-MB-231 lineage, *Colocasia esculenta*, GNA-related lectin, proteome profiling, nanoliposomes, antitumoral response

## Abstract

**Background/Objectives:** Tarin exhibits immunomodulatory and antiproliferative properties against several tumor cell lines. Nano-encapsulation in liposomes enhances its therapeutic potential by improving protein stability, bioavailability, and sustained release. Previous studies demonstrated that nano-encapsulated tarin induces cell cycle arrest, migration inhibition, apoptosis, and autophagy in triple-negative breast cancer cells; however, the molecular mechanisms underlying these effects remain poorly understood. To investigate the proteomic response elicited by nano-encapsulated tarin, MDA-MB-231 cells were treated for 24 and 48 h. **Methods:** Intracellular proteins were extracted, digested with trypsin, and analyzed by label-free LC-2D-MS/MS using HDMSE acquisition. Differentially expressed proteins were identified and quantified using the Progenesis QI platform, and then functional classification and pathway enrichment analyses were performed. **Results:** A total of 2818 proteins were identified, of which 2150 displayed time-dependent modulation following treatment. After 24 h, cells exhibited an adaptive stress response profile characterized by increased DNA repair proteins (*CHEK1*, *CDK12*), migration/remodeling factors (*LAMA4*, *CTTN*, *A2M*), and immune/cell cycle regulators (*PER2*, *HLA-B*), while antioxidant proteins (*SOD1*, *GPX1*) and BRCA1 were reduced, indicating oxidative stress and DNA damage. After 48 h, the proteomic profile shifted toward cell death, with increased *PARK7*, *OPA1*, *ATL3*, and CASP8 expression, disruption of DNA repair and cell cycle regulators (*CHEK1*, *CDK12*, *MSH6*, *KIF2C*), and decreased migration-related proteins (*LAMA4*, *CTTN*, *ITGB3*, *A2M*). **Conclusions:** Nano-encapsulated tarin promotes a time-dependent transition from early adaptive stress responses to apoptosis, autophagy, cell cycle disruption, and loss of migratory capacity. These findings provide novel insights into the molecular mechanisms underlying tarin antitumoral activity and support its potential as a promising therapeutic strategy against triple-negative breast cancer.

## 1. Introduction

Breast cancer is the most frequently diagnosed type of cancer among women worldwide and one of the leading causes of cancer-related deaths among this group [1]. Despite significant advances in diagnosis and therapeutic approaches, triple-negative breast cancer (TNBC) continues to represent a major clinical challenge due to its high aggressiveness, molecular heterogeneity, and absence of hormone and HER2 receptors, which limit targeted therapies. Patients presenting TNBC frequently experience early recurrence and low overall survival rates, reinforcing the need for new therapeutic strategies that combine antitumor efficacy and low systemic toxicity [2,3,4].

Breast cancer treatment depends on molecular subtype identification, which implies different therapeutic modalities. Concerning hormone-positive tumors (ER+/PR+), hormone therapy, such as application of tamoxifen, aromatase inhibitors, and ovarian suppression, represents one of the main approaches [5,6,7]. Regarding HER2-positive tumors, the use of monoclonal antibodies, i.e., trastuzumab, pertuzumab, and antibody–drug conjugates trastuzumab deruxtecan andT-DM1, has improved clinical management, increasing survival rates and antitumor response [5,8,9]. Cytotoxic chemotherapy drugs, including anthracyclines, taxanes, and cyclophosphamide, however, remain central in breast cancer treatments across multiple clinical settings. In parallel, immunotherapy with pembrolizumab has become an important component in TNBC management that is incorporated both in neoadjuvant regimens and in the treatment of metastatic disease [10,11]. Furthermore, PARP inhibitors such as olaparib are effective in patients presenting BRCA1/2 mutations, representing an advance in personalized therapy [12]. However, cumulative toxicity, therapeutic resistance, and limited efficacy against highly aggressive tumors still represent significant barriers, indicating the need for novel antitumoral compounds in innovative formulations.

Among natural bioactive compounds, food-plant lectins have emerged as promising molecules in this sense, due to their antitumor, immunomodulatory, and antiproliferative activities [13,14,15]. Tumor cells exhibit altered glycosylation patterns that are recognized by lectins that bind to specific glycans associated with invasion, metastasis, and immune evasion [16,17]. Recent studies have demonstrated that several lectins induce apoptosis, autophagy, and cell cycle arrest, as well as inhibit angiogenesis in breast tumors, also acting as diagnostic tools and targeted drug delivery systems [16,17,18,19]. Tarin, purified from *Colocasia esculenta* (L.) Schott, stands out as a relevant candidate in this regard, as it recognizes high-mannose and complex N-glycans, both widely expressed in tumoral and immune cells [20,21,22]. In addition to exhibiting biocidal and anti-inflammatory activities, tarin also displays a cytokine-mimetic effect on hematopoietic cells [23] while retaining cytotoxic activity when nano-encapsulated, also displaying the ability to cross complex biological barriers [24,25,26]. This combination of molecular specificity and anticancer potential makes tarin an attractive candidate for the development of innovative therapeutic formulations.

Nanotechnology has played a critical role in improving the efficacy and selectivity of antitumor agents. Liposomes are biocompatible spherical vesicles capable of encapsulating hydrophilic and hydrophobic drugs, allowing for controlled release and reduced systemic toxicity [27,28]. Pegylated liposomal doxorubicin (PLD; Doxil^®^/Caelyx^®^) is one of the most well-established carriers and has been used to treat metastatic breast cancer, displaying similar efficacy to the conventional formulation, but less cardiotoxicity [29,30]. Clinical trials have also demonstrated that PLD can be safely combined with trastuzumab and taxanes in the treatment of HER2-positive tumors, supporting its integration into combination regimens in targeted therapies [31]. Furthermore, novel formulations such as liposomal paclitaxel and, more recently, fulvestrant, have been tested against breast cancer, offering better stability and greater accumulation in the tumor but reduced adverse effects [32,33,34]. In this context, tarin liposome nano-encapsulation integrates a widely established oncological strategy to enhance novel therapeutic compounds, increasing stability, cellular internalization, and safety features.

In this context, tarin liposome nano-encapsulation significantly enhances this compound’s antitumoral activity, increasing stability, bioavailability, and efficient cellular internalization. While free tarin undergoes proteolytic degradation and exhibits low permeability, its liposomal formulation facilitates endocytosis mediated by mannose-rich glycans, abundantly expressed in tumor cells, promoting greater intracellular lectin accumulation [16,20,21,24]. In MDA-MB-231 adenocarcinoma cells, significant reductions in cell viability, caspase-3/7 activation, and autophagic vacuole formation, as well as morphological alterations, including organelle swelling and disintegration, have been reported [25]. Nano-encapsulated tarin also reduces tumor migration and maintains cytotoxic activity after crossing the in vitro blood–brain barrier [24,26]. Thus, nano-encapsulation not only protects tarin but also intensifies its cytotoxic effects, reinforcing its relevance as a candidate for innovative drug therapies against aggressive tumors such as TNBC.

Quantitative proteomics has emerged as an essential tool for understanding molecular mechanisms induced by antitumoral compounds. Advanced mass spectrometry techniques, such as LC-2D-MS/MS, make it possible to identify thousands of proteins simultaneously and map pathways associated with mitochondrial stress, metabolism, signaling, and cell death processes, among others [30,31]. The employment of proteomic analyses to follow the molecular events associated with cell death, migration, proliferation, immunogenicity, and adaptive responses to stresses may contribute to the understanding of cytotoxicity induced by tumor nano-therapies [33,34]. Thus, the temporal analysis of proteomes from MDA-MB-231 cells exposed to nano-encapsulated tarin may provide a unique opportunity to elucidate its mechanisms of action and advance the development of more specific TNBC therapies.

In this sense, this study aimed to investigate proteomic alterations induced by nano-encapsulated tarin on TNBC cells (MDA-MB-231), to elucidate molecular mechanisms associated with cytotoxicity and tumor cell death pathways triggered by this food-plant bioactive compound.

## 2. Materials and Methods

### 2.1. Tarin Purification

*Colocasia esculenta* (L.) Schott corms were purchased at a local market in Rio de Janeiro, Brazil, and a voucher taro plant specimen was deposited (RFA-39.962) at the RFA Herbarium (https://specieslink.net/col/RFA) belonging to the Biology Institute, Botany Department at the Federal University of Rio de Janeiro (RJ, Brazil). A crude taro extract was prepared as previously described [35] and purified through affinity chromatography by fast-performance liquid chromatography (FPLC) employing an Akta purifier 10 system (GE Healthcare, Chicago, IL, USA) coupled to a Cibacron Blue 3G-A Agarose column (Sigma-Aldrich Co., St Louis, MO, USA), which exploits dye–ligand interactions based on hydrophobic and ionic binding properties [20]. The obtained tarin fraction was freeze-dried (Liobras, São Paulo, SP, Brazil), and protein concentrations were estimated using a Total Protein Kit, Micro Lowry, Peterson’s Modification, employing bovine serum albumin (BSA) (Sigma-Aldrich Co.) as the external standard.

### 2.2. Tarin-Loaded Nanoliposome Preparation

Liposomes about 150 nm in size were prepared using the following lipid component mixture: DOPE (1,2-dioleoyl-sn-glycerol-3-phosphoethanolamine), MPEG 2000-DSPE 1,2-distearoyl-sn-glycero-3-phosphoethanolamine-N-[amino (polyethylene glycol)-2000] (Lipoid GMBH, Ludwigshafen am Rhein, Germany), and cholesteryl hemisuccinate (CHEMS) (5.7:3.8:0.5 µmol of lipids) (Sigma-Aldrich Co.), all dissolved in chloroform. After removing the organic solvent using a rotary evaporator (Buchi, São Paulo, SP, Brazil), the resulting thin lipid film was rehydrated in 0.3 M ammonium sulfate, pH 7.4, containing 1 mg/mL tarin, and stirred for 40 min at room temperature [24]. After incubation, the suspension was sonicated (130 W, 40 kHz, 1 min, 25 °C) and extruded for 12 cycles through a 0.2 µm membrane to reduce the size and ensure homogeneity. Ultracentrifugation (150,000× *g* by 90 min at 4 °C) (Beckman Coulter, Indianapolis, IN, USA) was performed to separate tarin-loaded liposomes (pellet) from non-encapsulated protein (supernatant). After ultracentrifugation, the supernatant containing residual ammonium sulfate and non-encapsulated tarin was carefully collected for protein quantification, while the liposomal pellet was resuspended in HEPES-buffered saline (HBS, pH 7.4) to obtain tarin nano-encapsulated formulation. The particle size of the liposomal pellet (~150 nm and polydispersity index < 0.2) was determined by dynamic light scattering (DLS). Nano-encapsulation efficiency was determined by the ratio between non-encapsulated tarin in the supernatant and the total amount of tarin at the beginning of the encapsulation process, measured using the aforementioned Total Protein Kit.

$$Encapsulation\;efficiency\;\left(\%\right)=\left[\frac{Loaded\;protein-Nonencapsulated\;protein\;(mg)}{Loaded\;protein\;(mg)}\right]\times 100$$

Ultracentrifugation was chosen based on the marked difference in sedimentation coefficients between ~150 nm nanoliposomes and free tarin and followed a protocol previously established by our group [24]. Although this indirect method does not exclude adsorption or co-pelleting of free tarin, which could slightly overestimate encapsulation efficiency, the values obtained are consistent with the low leakage rate and stable release profile previously reported [24], supporting the reliability of the quantified encapsulated tarin fraction.

### 2.3. Human Cell Lines and Culture Conditions

The human mammary adenocarcinoma cell line MDA-MB-231 (ATCC HTB-26) was obtained from the Rio de Janeiro Cell Bank (BCRJ, Rio de Janeiro, RJ, Brazil), available at https://bcrj.org.br/. Cells were cultured in high-glucose Dulbecco’s Modified Eagle Medium (DMEM) supplemented with 10% fetal bovine serum (FBS) (Gibco Life Technologies, Grand Island, NY, USA) at 37 °C in a humidified atmosphere containing 5% CO_2_.

MDA-MB-231 cells were seeded at a density of 1.0 × 10^6^ cells/mL in 75 cm^2^ culture flasks until they reached a semi-confluent layer and then detached using the TrypLE™ Express enzyme (Gibco Life Technologies) for 5 min at 37 °C. Following centrifugation at 200× *g* for 10 min, the cells were transferred to 24-well plates at a density of 1.5 × 10^5^ cells/well and incubated for 24 h to allow adherence.

After this initial incubation, the culture medium was replaced with a fresh one containing nano-encapsulated tarin at 72 μg/mL (ET). Cells were incubated for 24 h or 48 h, resulting in two experimental groups: ET24h and ET48h. Additionally, untreated cells were incubated under the same conditions for 24 h and 48 h and served as controls (C24h and C48h). Cells were collected for downstream analyses at the end of the respective incubation periods.

### 2.4. Intracellular Protein Extraction and Digestion

Each experimental cell group, treated or control, described above was adjusted to 1 × 10^6^ cells/mL. After centrifugation at 10,000× *g* for 5 min, cells were resuspended in 50 μL of a 50 mM ammonium bicarbonate buffer, pH 8.0, and then cell lysing was carried out employing five freeze–thaw cycles in liquid nitrogen intercalated by 30 s sonication intervals in an ultra sonicator bath at 50 watts. Protein quantification was performed using the BCA method performed with the Total Protein Kit (Sigma-Aldrich Co.). Then, 100 μg of protein from each sample was added to 25 μL of the RapiGest^TM^ surfactant reagent 0.2% (*w*/*v*) (Waters Co., Milford, MA, USA) and heated at 80 °C for 15 min. The protein extract was treated with a dithiothreitol solution (DTT) 5 mM (Sigma-Aldrich Co.) and incubated for 30 min at 60 °C. Alkylation was carried out by adding a 14 mM iodoacetamide solution (Sigma-Aldrich Co.) followed by 30 min incubation at room temperature in the dark. Protein digestion was performed equally for all samples by the addition of 2 μg of a trypsin solution (Promega Co., Madison, WI, USA) at a 1:50 ratio and incubation for 14 h at 37 °C. To remove the RapiGestTM, a 90 min incubation period at 37 °C in 20 μL of a 5% (*v*/*v*) trifluoroacetic acid (TFA) solution was carried out, followed by centrifugation at 16,000× *g* for 30 min at 4 °C.

After digestion, the peptide mixture was dried thoroughly in a vacuum concentrator (Savant™ SpeedVac SPD120 (Thermo Fisher Scientific, MA, USA)), resuspended in 500 μL methanol at 5%, and purified employing an OASYS SPE HLB (Waters Co., MA, USA) following the manufacturer’s instructions. The obtained peptides were resuspended in a solution containing 0.1% formic acid and 3% acetonitrile and stored at −30 °C until protein identification and quantitation by LC-MS/MS employing a Synapt XS mass spectrometer (Waters Co.).

### 2.5. Mass Spectrometry Analysis

Trypsinized peptide mixtures from treated and non-treated samples were analyzed on a SYNAPT XS instrument coupled to an ACQUITY UPLC M-Class System with 2D Technology (Waters Co.) using a two-dimensional separation reversed-phase column. The ion mobility data-independent mode of acquisition (HDMS^E^) label-free quantitative expression method was applied.

Instrumental parameters for electrospray ionization mass spectrometry (nanoESI) were optimized on the tune page. Detector sensitivity was calibrated with 100 pg/μL leucine enkephalin (Le-enkephalin 556.277 Da). Mass-to-charge (*m*/*z*) calibration was performed using 100 fmol/μL Glu-fibrinopeptide B (785.843 Da) through the MassLynx system (IntelliStart) in resolution mode. The final acquisition parameters were as follows: capillary voltage: 3 kV, sample cone voltage: 35 V, extraction cone voltage: 4 V, source temperature: 80 °C, cone gas flow: 10 L/h, nano-flow gas: 0.5–0.6 bar, and purge gas: 600–800 L/h. All analyses were conducted in positive ionization mode. Mass spectra data were acquired over an *m*/*z* range of 50–2000 Da with a scan time of 1 s. The ramped transfer collision energy was set between 15 and 50 V, with a total acquisition time of 75 min.

Prior to the LCMS/MS analysis, all samples were resuspended in 40 µL of 20 mM ammonium formate, pH 10, to a final peptide concentration of 1 µg/µL. A total of 5 µg of digested protein (1 µg/µL) from one biological replicate was analyzed in two technical replicates, with 5 µL of the sample injected onto the 1D column (nanoEase M/Z^TM^ Peptide BEH C18, 130 Å, 5 µm, 300 µm × 50 mm column) in each replicate. The peptides were fractionated into six step gradients in the trapping phase according to the acetonitrile (ACN) concentration, at 11, 14, 17, 20, 50, and 80% for 9.50 min each trapping step, comprising 0.5 min in 97% A and 3% B; 0.5 to 1.0 min in 11, 14 17, 20, 50, 80% A and 89, 86, 83, 80, 50, 20% B (respectively, for each step); 1.0 to 4.0 min maintained in 11, 14 17, 20, 50, 80% A and 89, 86, 83, 80, 50, 20% B; 4.0 (respectively, for each step) to 4.5 min in 97% A and 3% B; and 4.5 to 9.5 min maintained in 97% A and 3% B, for re-equilibration of the 1D column. Following the trapping step, the system was maintained under isocratic conditions using a mobile phase consisting of 97% A and 3% B for 75 min at a flow rate of 2 µL/min.

Peptides eluted from the 1D column were captured using an Acquity UPLC M-Class Symmetry C18 Trap column (Waters Co., Milford, MA, USA) (100 Å, 5 µm, 180 µm × 20 mm). Finally, the eluted peptides from the trap column were separated on a reversed-phase Acquity UPLC M-Class HSS T3 C18 analytical column (75 µm × 150 mm) applying a linear gradient performed by the lower binary pump (lower BSM) at a 0.4 µL/min flow rate consisting of 0.1% formic acid in water (mobile phase A) and 0.1% formic acid in ACN (mobile phase B) for 75 min. The gradient consisted of 1.66 min in 93% A and 7% B, 1.66 to 51.36 min in 60% A and 40% B, 51.36 to 53.02 min in 15% A and 85% B, 53.02 to 57.02 min in 15% A and 85% B, and 57.02 to 57.99 min in 93% A and 7% B, and then column re-equilibration (93% A and 7% B) from 57.99 to 75 min. Both gradient programs corresponding to the 1D and 2D columns are graphically represented in Supplementary Material Figure S1.

### 2.6. Bioinformatics Mass Spectra Data Analysis and Protein Quantification

The raw mass spectrometry data files were imported to and processed by Progenesis QI for proteomics, software version 4.1 (Nonlinear Dynamics, Waters Co.), for label-free quantification. The software was used to align all chromatogram runs based on the retention time to a reference run automatically selected by the algorithm to maximize overall alignment quality. Following alignment, feature detection was carried out, and features with charge states between +1 and +10 and at least one isotope were retained for further analysis. Normalization of peptide intensities across runs was performed using the default global normalization method provided by Progenesis to correct for systematic variations of up to a factor of 2 compared to the reference chromatogram. Protein identification was carried out by searching against the human reference proteome database (UP000005640) from UniProtKB (https://www.uniprot.org/) containing reviewed and unreviewed annotations (accessed 25 November 2024, release June 2024). The search parameters comprised two missed cleavages by trypsin, carbamidomethyl (C) as fixed modifications, and methionine oxidation as variable modifications. The precursor and fragment ion mass error tolerances were adjusted to default values. The positive protein match criteria were at least two fragment ions per peptide, five fragment ions per protein, and at least one peptide per protein hit. A false-positive discovery rate of up to 1% was allowed. The algorithm tool estimated the protein relative quantification ratio between the data from treated and non-treated samples.

The resulting search files from each of the chromatogram fractions (11, 14, 17, 20, 50, and 80%, respectively) were then reimported into Progenesis and combined for peptide-to-feature assignment and protein inference. All peptides were considered for relative protein quantification. Statistical analyses concerning differential protein abundance were conducted using the built-in ANOVA test within Progenesis, considering a *p* value < 0.05 as significant. All peptides with a Progenesis score of less than 5.0 were eliminated in the final protein identification stage.

Fold changes were calculated based on normalized abundance ratios between experimental groups. Proteins were considered differentially expressed if they exhibited a relative fold change >1 and <−1 with a significance threshold of *p* < 0.05 determined after the *t*-test. Volcano plots were designed with Log_2_fold change on the *x*-axis versus the −Log_10_ of the significance threshold on the *y*-axis. Data visualization and interpretation were supported by a Principal Component Analysis (PCA), hierarchical clustering, and volcano plots, generated by exporting the PROGENESIS CSV data for processing in external tools such as the VolcaNoseR, [36] web app, used to create, explore, label, and share volcano plots.

### 2.7. Statistical Analyses

All experiments were performed in triplicate, and the results are expressed as means and standard deviations. Data were compared by a one-way or two-way Analysis of Variance (ANOVA) followed by multiple comparisons using Tukey’s post hoc test or the multiple *t*-test followed by the Holm–Sidak method post-test. GraphPad Prism version 7 (GraphPad, San Diego, CA, USA) was used for all statistical analyses, considering *p* < 0.05 as significant.

## 3. Results and Discussion

### 3.1. Total Intracellular Protein Quantification Following Cell Treatment with Tarin-Loaded Nanoliposomes

The protein profiles from MDA-MB-231 cells treated with nano-encapsulated tarin (ET) were quantified and identified by LC-2D-MS/MS. In our previous study, we demonstrated that empty nanoliposomes do not interfere with the antitumor effect of nano-encapsulated tarin. Based on this and on the extensive data generated in the present study, we have chosen to focus on the proteome dataset of cells treated with nano-encapsulated tarin. Similarly, free tarin was shown to exhibit an antitumoral effect that, most of the time, was inferior to that of the nano-encapsulated tarin [25].

**Figure 1:**
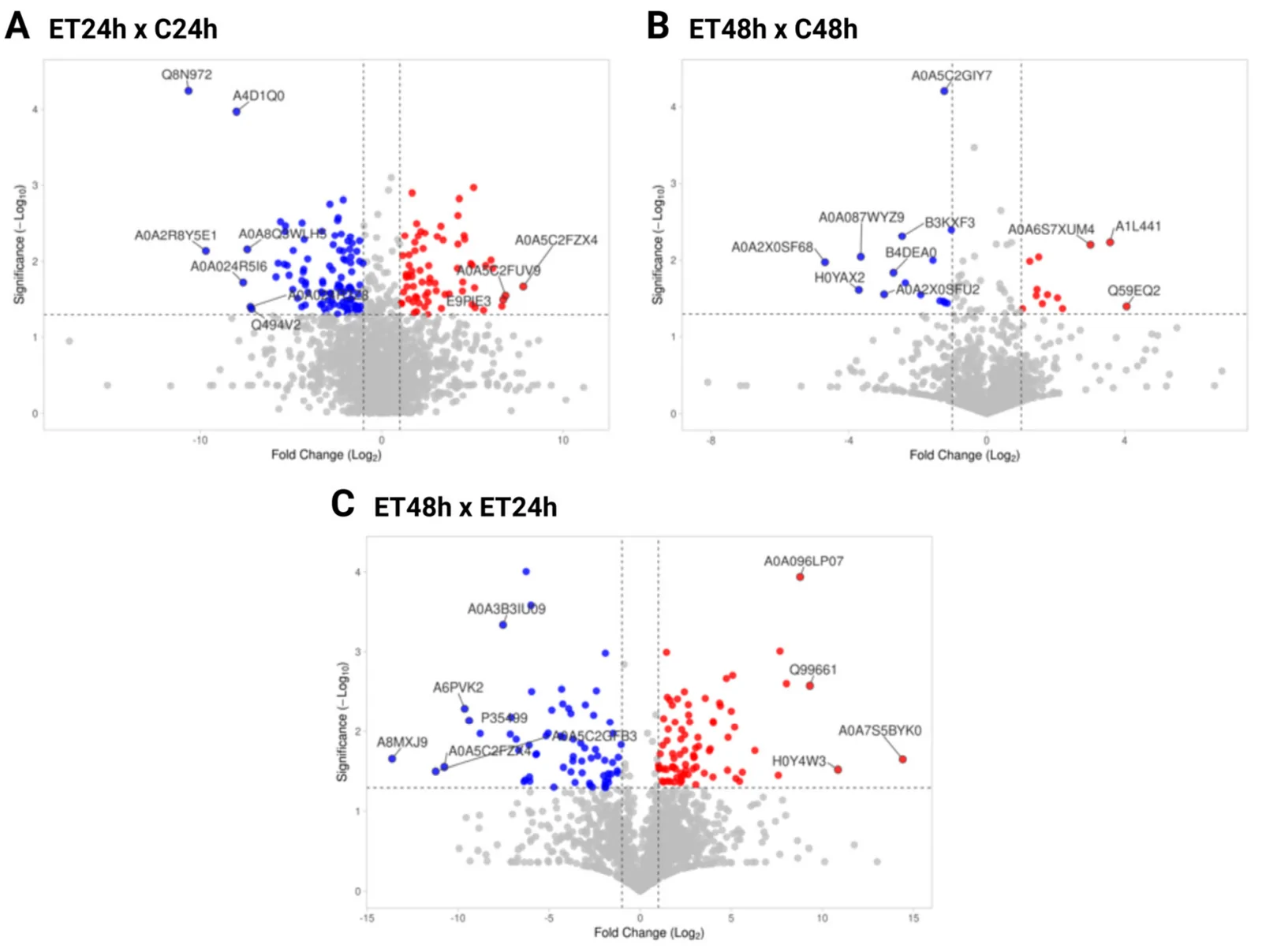
Volcano plots plotted using VolcaNoseR including all expressed proteins in the ET24h × C24h (**A**), ET48h × C48h (**B**), and ET48h × ET24h (**C**) groups. Red circles indicate proteins displaying increased fold change rates, while blue circles denote those proteins displaying decreased fold changes. Proteins that did not reach the significance threshold are represented in gray. A complete list of all up-/downregulated proteins is available in Supplementary Table S2. ET—cells exposed to nano-encapsulated tarin for 24 h or 48 h, C—non-treated cells.

A total of 2818 proteins were identified across all samples, and the complete list is provided in Supplementary Table S1. Of these, 2150 proteins showed differential abundance when comparing experimental groups after the 24 h and 48 h treatments. To analyze statistically significant changes in protein abundance, volcano plots were generated to evaluate the early impact of nano-encapsulated tarin on protein expression compared to untreated control groups (ET24h vs. C24h and ET48h vs. C48h), as well as to assess temporal changes in the response to tarin treatment over 48 h (ET48h vs. ET24h) (Figure 1A–C). These comparative analyses revealed distinct protein regulation patterns at each time point, indicating that both the presence of nano-encapsulated tarin and tarin exposure time can influence the proteomic profiles of treated cells (Table S2).

Among differentially expressed proteins, a total of 171 were found to be differentially expressed when comparing the ET24 h to the C24 h conditions, 69 of which were upregulated (Figure 1A, red circles) and 102 downregulated (Figure 1A, blue circles). In the comparison between the ET48 h and C48 h conditions, 27 differentially expressed proteins were identified, 12 of which were upregulated and 15 downregulated (Figure 1B, red and blue circles, respectively). The proteome comparison between the ET24 h and ET48 h conditions revealed 133 differentially expressed proteins, 72 of which were upregulated (Figure 1B, red circles) and 61 downregulated (Figure 1B, blue circles). The complete list of up- and downregulated proteins is provided in Supplementary Table S2.

The up-/downregulated proteins and their respective fold changes (Log 2) comparing the ET24 h vs. C24h, ET48 h vs. C48 h, and ET48h vs. ET24h groups are presented in Table S2. Among them, the immunoglobulin heavy-chain protein IGH c823_heavy_IGHV1-2_IGHD3-10_IGHJ4 (A0A7S5BYK0) showed the highest upregulation, with a fold change of 14.4 in the ET48 h vs. ET24 h comparison. This higher protein abundance in MDA-MB-231 cells following nano-encapsulated tarin treatment may indicate an adaptive response of tumor cells to tarin-induced stress. Studies have demonstrated that breast cancer cells, including MDA-MB-231 cells, can express immunoglobulins like IGHG1, which contributes to cell proliferation, apoptosis evasion, and cancer therapy resistance [37,38,39,40]. It is also associated with the activation of PI3K/AKT and MEK/ERK signaling pathways, contributing to the aggressiveness of the triple-negative phenotype [41]. Thus, the IGH c823 upregulation may reflect the compensatory mechanism for cell survival commonly observed in aggressive tumors when they are subjected to the therapeutic stress.

On the other hand, the Tweety Homolog 3 (TTYH3-A8MXJ9) protein presented the most significant downregulation in MDA-MB-231 cells treated with nano-encapsulated tarin (ET48 h vs. ET24 h). This protein functions as a calcium-activated chloride channel involved in cell volume, ionic homeostasis, and Ca^2+^ influx regulation [42,43]. Studies have demonstrated that its overexpression in solid tumors, such as in hepatocellular carcinoma, is associated with tumor development and progression through the MK5/GSK3-β/β-catenin signaling pathway, promoting cell proliferation, migration, invasion, and apoptosis inhibition [44]. Based on this, the reduction in TTYH3 abundance may suggest a molecular mechanism underlying tarin action that suppresses pro-survival and migratory pathways.

In addition, previous studies indicate that treatment with nano-encapsulated tarin induces mitochondrial stress/swelling in MDA-MB-231 cells, an event that could be triggered by mitochondrial calcium overload [25]. The observed drop in TTYH3 expression may contribute to this effect by compromising the ability of cells to control cytosol calcium levels, allowing for its accumulation and subsequent mitochondria uptake and thereby leading to programmed cell death activation [45,46]. Thus, data reported herein may indicate that TTYH3 downregulation could enhance the cytotoxic action of tarin via calcium-dependent mitochondrial destabilization. However, further studies need to confirm these interpretations by targeting functional assays in the future.

### 3.2. Gene Classification According to Molecular Function

**Figure 2:**
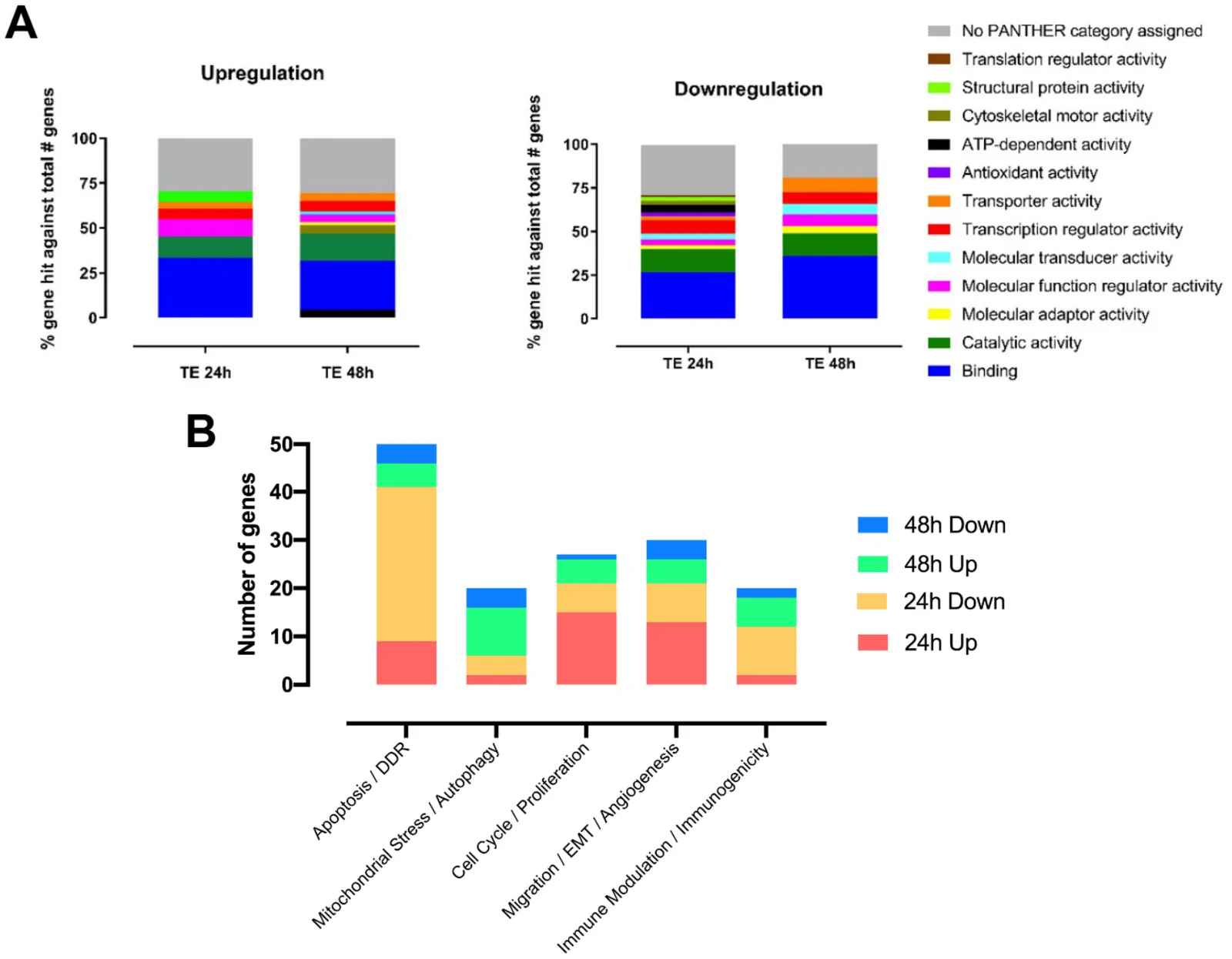
Total gene distribution classified by category and temporal expression profiles. Proteomic analyses were conducted in protein extracts obtained from MDA-MB-231 cells following 24 h/48 h treatment with nano-encapsulated tarin. Protein-encoding genes were grouped according to their cellular molecular activity (**A**) and the anticancer mechanisms they are involved in (**B**). DDR—DNA damage response; EMT—epithelial–mesenchymal transition.

The molecular functions of the differentially expressed proteins following nano-encapsulated tarin treatments were assessed using pantherdb.org, an extensive curated biological database of gene protein families used to classify and identify gene products (Figure 2A). A significant portion (36.7%) of the proteins was associated with binding activity, which suggests a relevant role in protein–protein interactions and in the modulation of supramolecular complexes [47]. Of the proteins, 20.4% displayed catalytic activity, indicating their involvement in biochemical reactions and cell metabolism. Although additional functions were identified, such as transcriptional regulators (8.2%), the *binding* and *catalytic activity* categories were predominant. Strande, Canelle [40] reported similar results when analyzing the MDA-MB-231 cell proteome, further reinforcing the relevance of these protein functions to tumors, and highlighting potential links to their growth and maintenance.

**Figure 3:**
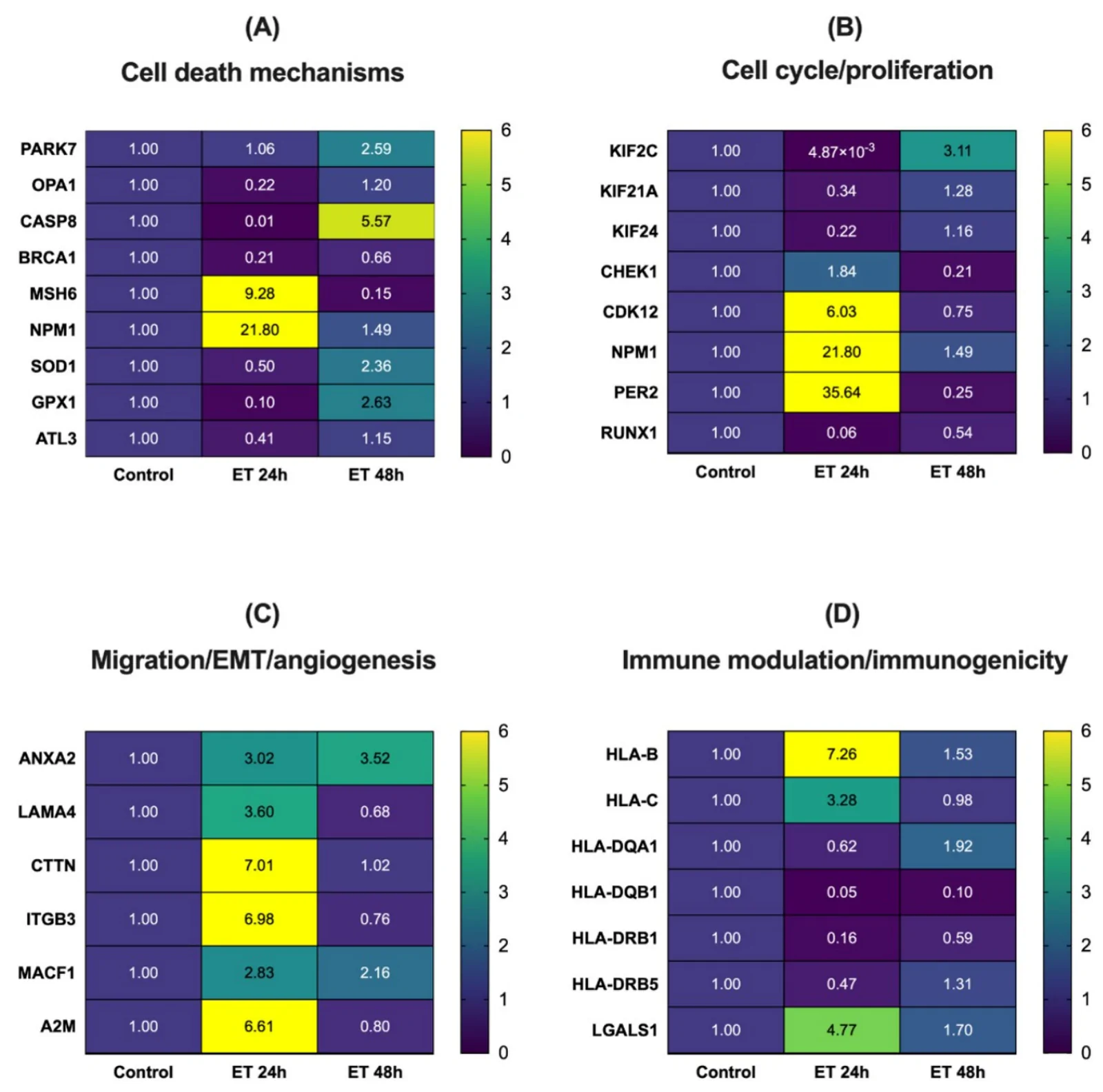
Heatmap representing protein expression dynamics in MDA-MB-231 human adenocarcinoma cells treated with nano-encapsulated tarin for 24 h (ET24h) and 48h (ET48h). Each heatmap cell displays the fold change value, while the intensity of each color reflects up- or downregulation of gene expression according to the scale on the right (light tones indicate higher expression and dark tones lower expression). Control—non-treated cells at 24 h (C24h); ET—cells exposed to nano-encapsulated tarin for 24 h or 48 h.

Proteins displaying binding functions play a central role in the formation of regulatory and signaling complexes, such as the death-inducing signaling complex (DISC), which activates caspase-8, triggering apoptosis [48]. Proteins displaying catalytic activity, such as caspases and lysosomal proteases (cathepsin D), participate directly in the cleavage of targets that execute programmed cell death [49,50]. The profile of genes whose expression is regulated by tarin suggests that this lectin can modulate both protein–protein interactions and the enzyme-regulated reaction essential in apoptosis induction, serving as a basis for the investigation of cell death mechanisms. Indeed, when protein-coding genes were analyzed, based on their involvement in anticancer mechanisms, five essential cancer hallmarks were detected, suggesting that tarin exhibits a broad antitumoral spectrum (Figure 2B). Tarin-loaded nanoliposomes can dynamically and temporally up- and downregulate gene expression. Most of these genes integrate cell death mechanisms that include apoptosis and/or autophagy activation, DNA damage responses, or mitochondrial stress. The downregulation of several genes associated with apoptosis/DDR following 24 h exposure to nano-encapsulated tarin suggests a compensatory mechanism that adapts to initial stressful conditions. The initial response culminates in cell death, which is marked by the late expression of genes involved in mitochondrial stress and autophagy mechanisms after 48 h. A similar dynamic expression of genes involved in proliferation and metastatic potential has also been detected in MDA-MB-231 cells, reinforcing these findings [25] (Figure 2B). Proteins involved in immune modulation and immunogenicity were also modulated by nano-encapsulated tarin. Notably, molecules related to antigen presentation, including HLA-B and HLA-C (MHC class I) and HLA-DQA1, HLA-DQB1, HLA-DRB1, and HLA-DRB5 (MHC class II), were differentially expressed (Figure 3D). These proteins regulate essential mechanisms that can enable cancer cells to be recognized by T lymphocytes, under interaction with major histocompatibility complex I and II receptors (MHC I and II) involved in antigen presentation [51,52].

In a previous study, we showed that the mechanisms underlying nano-encapsulated tarin-induced MDA-MB-231 cell death involve both apoptotic and autophagic pathways. These include activation of the caspase-3/7 cascade, cell cycle arrest, and the formation of acidic vacuolar organelles, which are characteristic of autophagosomes [25]. Here, the previous findings are reinforced by the identification of those proteins, identified through their respective encoding genes, that are involved in the antitumoral response triggered in MDA-MB-231 cells (Figure 3).

Based on these findings, a heatmap was generated to depict the expression levels of the most relevant genes related to four antitumoral mechanisms categories, revealing the impact of nano-encapsulated tarin exposition on human breast cancer cells (Figure 3). In this sense, the representative genes *PARK7*, *ANXA2*, *CASP8*, *CHEK1*, and *HLA-B* play central roles in maintaining cell homeostasis, controlling the cell cycle and migration capacity, and regulating cell death and immunogenicity.

**Figure 4:**
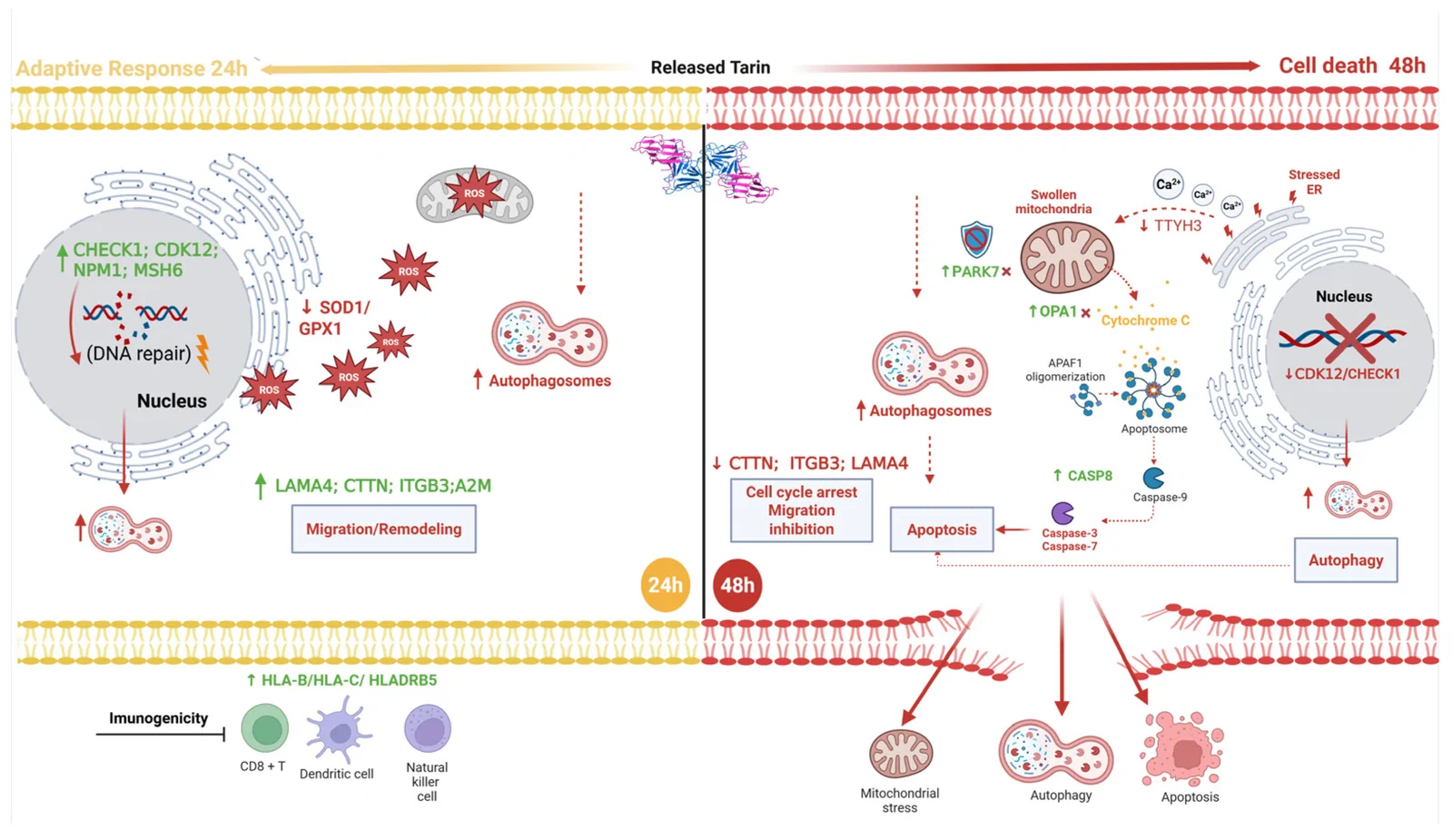
Proposed model of time-dependent cellular responses induced by nano-encapsulated tarin in MDA-MB-231 cells. At 24 h (**left**), cells activate adaptive stress mechanisms, with increased ROS, DNA damage, and induction of repair, survival, and migration. At 48 h (**right**), this adaptation fails, resulting in DNA repair collapse, cell cycle arrest, and activation of autophagy and apoptosis, culminating in cell death. Proteins shown in green are upregulated, whereas proteins in red are downregulated. Figure created in BioRender Conte-Junior, C. A. (2026) https://BioRender.com/663dyl6.

Figure 3 suggests that cancer cells undergo oxidative stress upon first contact with nano-encapsulated tarin, evidenced by the depletion of antioxidant proteins encoded by *SOD1* and *GPX1* genes. In this scenario, the accumulation of reactive oxygen species (ROS) can cause DNA damage, triggering the upregulation of proteins involved in DNA repair at 24 h, such as *CHEK1*, *CDK12*, *NPM1*, and *MSH6*, while *BRCA1* decreases and fails with DNA repair (Figure 3A,B). At the same time, genes associated with pro-migratory function, especially *AXN2* and *LAMA4*, are upregulated in 24 h as a compensatory mechanism of cell survival (Figure 3C). This scenario indicates cellular attempts to withstand DNA damage and maintain genomic integrity, consistent with the proposed lectin mechanism of binding to surface carbohydrates followed by stress signaling [53,54,55,56,57]. However, this excessive response seems to be insufficient, culminating in cell death through autophagy and apoptosis activation, which was previously reported for nano-encapsulated tarin in our previous study [25]. In fact, gene expression at 48 h reveals irreversible cell death commitment, characterized by the DNA repair response collapse (reduced *CHEK1*, *CDK12*, and *MSH6*), mitochondrial stress and autophagy activation (increased *PARK7*, *OPA1*, *ATL3*), cell cycle impairment (reduced *CHEK1* and *PER2*, increased *KIF2C*), and compromised migration capacity (reduced *LAMA4*, *CTTN*, *ITGB3*, and *A2M*), indicating a transition from early adaptive responses to irreversible cell death. To better illustrate these time-dependent cellular responses, a schematic model was developed (Figure 4).

Increases in PARK7 (DJ-1), OPA1, and ATL3 expressions, which include proteins essential to mitochondrial stress and autophagy regulation, were observed after 48 h (Figure 3A). *PARK7/DJ-1*, a multifunctional protein that protects against oxidative stress, maintains mitochondrial integrity and regulates autophagy/mitophagy [58,59]. *PARK7/DJ-1* is able to interact with the PINK1/Parkin axis, modulating the removal of damaged mitochondria, and also inhibiting excessive autophagy, favoring cell survival under stress conditions through multiple cell survival pathways, including the PI3K/Akt/mTOR and HIF-1α pathways [59,60,61]. Here, the increase in *PARK7* expression after 48 h exposure to tarin may represent a late attempt to adapt to oxidative stress induced by decreased *SOD1* and *GPX1* at 24 h [61,62]. Considering our previous study reported the activation of autophagy by nano-encapsulated tarin, the increase in *PARK7* expression after 48 h may indicate that this protein was insufficient to prevent autophagic cell death [25,61]. Similar findings have been noted for other cell cultures, such as prostate cancer PC3 and LNCaP cell lineages [63]. In these cases, treatment by retigeric acid B (RAB) prompted slightly increased *PARK7/DJ-1* expression before 6 h exposure followed by a marked decrease at 24 h, which, in turn, triggered oxidative stress and pronounced mitochondrial swelling and then promoted cell mitophagy [63].

*OPA1*, a GTPase located in the inner mitochondrial membrane, plays a crucial role in mitochondrial fusion and in the maintenance of crest structure, preserving bioenergetic integrity and apoptosis regulation [64,65]. Here, a significant reduction in *OPA1* expression following 24 h of treatment was observed (Figure 3A), which could lead to decreased mitochondrial fusion and possible fragmentation induction. The downregulation of *OPA1* has consistently been associated with mitochondrial fragmentation and apoptosis in hepatocellular carcinoma cells (Huh7 and LH86) exposed to sorafenib [65]. In addition, targeting *OPA1* inhibition has been effective in reducing the growth of TNBC, both in vitro and in vivo, suggesting a promising therapeutic strategy against these tumors [66]. On the other hand, *OPA1* expression increased at 48 h, shown herein, which may represent an adaptive response, preventing the release of mitochondrial cytochrome c and the activation of caspase 3 (Figure 4). However, considering the exposed cells are under severe stress, the protective capacity of this GTPase may not be enough to maintain cell viability, corroborating our previous results, which indicated mitochondrial swelling and cell death at 48 h after nano-encapsulated tarin treatment [67,68,69]. These findings reinforce that the time-dependent response of *OPA1* and *PARK7* may represent a dynamic mitochondrial response axis to the stress induced by nano-encapsulated tarin, where the balance between fusion and antioxidant protection is progressively lost under prolonged lectin exposure. Moreover, the 48 h concomitant increase in *ATL3* (atlastin-3), an ER-phagy receptor, indicates the activation of a cellular quality control mechanism that eliminates damaged ER under stress conditions. The induction of ER-phagy is consistent with our previous study, which reported ER enlargement and the frequent engulfing of autophagosomes containing ER following nano-encapsulated tarin treatment, suggesting that ER stress contributes to the cytotoxic response [25,70,71,72].

Here, *CASP8* expression was increased (5.57-fold) after 48 h of nano-encapsulated tarin treatment, suggesting a time-dependent pathway activation response (Figure 3A). *CASP8* plays a central role in the extrinsic apoptosis pathway through death receptor signaling, promoting cleavage and subsequent activation of executor caspases, such as caspase-3 and 7, and resulting in structural protein degradation and cell death [50,73,74]. Additionally, caspase-8 can cleave BID, linking extrinsic and intrinsic apoptotic pathways [75]. This pattern is consistent with our previous studies, in which nano-encapsulated tarin was shown to induce caspase-3/7 activation and cell death in MDA-MB-231 cells [25]. These findings may indicate that, following an initial mitochondrial stress and an insufficient adaptive response phase characterized by *OPA1* and *PARK7* downregulation (Figure 3A), a transition to the execution of the apoptotic program mediated by *CASP8* is elicited, culminating in cell death at later time points.

The time-dependent response observed for *CASP8* (Figure 3A) suggests a two-phase (early and late) cellular response pattern to the nano-encapsulated tarin. At 24 h, comprising the initial phase, the absence of detectable *CASP8* activation indicates that cells are still predominantly involved in mitochondrial defense and adaptation mechanisms, which are potentially mediated by *OPA1* and *PARK7*, seeking to restore redox balance and preserve structural crest integrity. However, prolonged/late exposure for 48 h seems to deplete this adaptive capacity, resulting in mitochondrial homeostasis disruption and extrinsic apoptosis pathway activation evidenced by an increase in *CASP8* expression, as illustrated in Figure 4. This pattern is observed when persistent stress converts pro-survival responses into pro-death signals, functioning as an irreversible stimulus for the activation of executor caspases and progression to programmed cell death [50,76].

Oxidative stress *status* and the subsequent DNA damage repair mechanism activation were noted to affect cell cycle progression (Figure 3B). The observed increases in *CHEK1* and *CDK12* at 24 h followed by decreases at 48 h indicate a transition from checkpoint activation to irreversible cell cycle dysregulation. *CHEK1* expression displayed a time-dependent pattern, with a 1.84-fold increase at 24 h followed by a pronounced decrease to 0.21-fold at 48 h (Figure 3B). *CHEK1* is a central DNA damage response regulator, activated primarily by the ATR pathway, and its initial induction at 24 h suggests that cells treated with nano-encapsulated tarin could sense genotoxic stress and activate DNA repair checkpoint mechanisms. However, the decrease noted at 48 h (Figure 3B and Figure 4) may indicate failure to maintain this response, possibly due to loss of repair ability and activation of proapoptotic pathways. After 48 h, as demonstrated in our previous study, the nano-encapsulated tarin treatment induced significant changes across all cell cycle phases characterized by cell accumulation in the G0/G1 phase and consequent decreases in the S and G2/M phases. The G0/G1 arrest impairs cell cycle progression and inhibits cell proliferation [25]. These data suggest that delayed *CHEK1* reduction may be associated with the inability to resume cell cycle progression, resulting in transition towards programmed cell death.

Moreover, the observed increased *PER2* gene expression at 24 h is consistent with antitumoral results (Figure 3B). This gene is a circadian clock gene that plays a tumor suppressor role through the regulation of cell cycle genes, including cyclins (A2, B1, D1, CDK4, CDK6, p53, p16, and p21), CDKs, and CDK inhibitors [77,78]. The *PER2* decrease at 48 h (Figure 3B), on the other hand, may paradoxically suggest cell cycle dysregulation, although, considering the exposed cells are already committed to apoptosis, this reduction may reflect a general disintegration of cell cycle control mechanisms. The increased *KIF2C/MCAK* expression at 48 h (Figure 3B) may contribute to antiproliferative activity, as this gene exhibits microtubule-depolymerizing activity and is critical for microtubule dynamics during the metaphase and anaphase, ensuring faithful chromosomal segregation. Increased *KIF2C* expression may result from tarin-induced chromosomal instability, consistent with mitotic errors leading to cell death [79].

These results are consistent with our previously reported data, in which MDA-MB-231 cells treated with nano-encapsulated tarin underwent cell death via both apoptosis and autophagy, evidenced by transmission electron microscopy (TEM) analyses, which revealed autophagosome accumulation [25]. Here, the gene expression dynamics observed in the heatmap (Figure 3) reinforce the hypothesis that nano-encapsulated tarin triggers a multifaceted and time-dependent cellular response which begins with adaptative mechanisms performed through protective responses mediated by *PARK7* and autophagy modulation during prolonged nano-encapsulated tarin exposure. This adaptive response can be overcome by irreversible mitochondrial function impairment (via *OPA1*), apoptotic pathway activation (*CASP8*), and cell cycle disruption via *CHEK1* modulation, making tumor cells pass from a state of attempted survival to irreversible cell death (Figure 4).

The migration capacity of MDA-MB-231 cells was also compromised following treatment with nano-encapsulated tarin at 24 h and 48 h, as we have reported previously and corroborate here through proteomic analyses (Figure 3C) [25]. The *ANXA2* gene encodes annexin A2, a multifunctional protein located on the cell surface that acts as a co-receptor for plasminogen and tissue plasminogen activator (tPA), facilitating plasmin activation and extracellular matrix degradation. In hepatocellular carcinoma cells, *ANXA2* promotes migration and invasion through the traffic regulation of microvesicles containing CD147, which induce the production of matrix metalloproteinase-2 (MMP-2) by fibroblasts [80,81]. Increased *ANXA2* expression in breast cancer cells is also associated with high metastatic capacity.

*LAMA4* expression, an essential extracellular matrix component that affects cell adhesion and migration, was reduced after 48 h exposition to nano-encapsulated tarin (Figure 3C). Its overexpression has been associated with tumor progression and metastases in several cancers, including breast adenocarcinoma, while its inhibition significantly reduces tumor cell migration and invasion [82]. Similarly, *CDK12* (cyclin-dependent kinase 12) regulates the transcription of genes involved in DNA repair and the cell cycle, and its dysregulation is associated with therapeutic resistance and the maintenance of tumor viability [83]. The observed decrease in *CDK12* levels after 48 h (Figure 3C) may indicate interruption of pro-survival gene transcription and loss of cell cycle control, both of which are compatible with the activation of apoptotic pathways, as reported previously by our group [25]. The reduction of cortactin (*CTTN*) at 48 h reinforces the anti-invasive potential of tarin, since this protein regulates actin polymerization, which is essential for the formation of invadopodia, i.e., cell membrane protrusions, used to degrade the extracellular matrix and invade surrounding tissues [66,84].

The regulation of human leukocyte antigen (*HLA*) expression reveals a dynamic immune response regarding tumor immunogenicity. *HLA* class I (*HLA-A*, *HLA-B*, *HLA-C*) and II (*HLA-DQA1*, *HLA-DQB1*, *HLA-DRB1*, *HLA-DRB5*) genes displayed varied and, in some cases, contradictory expression patterns, with increases and decreases in different alleles at both times (Figure 3D and Table S2). *HLA* class I expression allows for the presentation and recognition of tumor antigens by cytotoxic CD8+ T lymphocytes (CTL), and its downregulation is an escape strategy employed by tumor cells to prevent CTL-mediated cytotoxicity [85]. In breast cancer, *HLA* class I expression is associated with immune cell infiltration, which allows for antitumoral responses, culminating in a positive prognosis, while the downregulation leads to poor prognoses [86,87]. Here, the upregulation of *HLA-B* and *HLA-C* following 24 h of treatment (Figure 3D and Figure 4) may suggest that tarin could improve immunogenicity-related pathways, possibly increasing the capacity for antigen presentation and intercellular communication in tumor cells. Similarly, *HLA-DQA1/DRB5* upregulation may suggest enhanced antigen presentation to CD4+ helper T cells, which could facilitate antitumoral responses. TCD4+ cells can act through direct cytotoxicity by releasing perforin/granzyme molecules, the secreting cytokines, i.e., IL-2, IFN-γ, and TNF-α, responsible for macrophage and natural killer (NK) cell activation, or by intermediating the priming, expansion, and maintenance of effector cytotoxic T lymphocytes with better cytotoxic capacity and memory, contributing to better immunotherapy responses [88,89,90,91].

The heterogenous expression of different *HLA* alleles (Table S2) may reflect cellular attempts to modulate antigen presentation in response to tarin-induced stress. To confirm the aforementioned hypothesis that nano-encapsulated tarin improves MDA-MB-231 immunogenicity, additional studies should be conducted, employing flow cytometry for surface *HLA* class I and II, co-culture assays with T cells, immunohistochemistry assessments, or functional antigen presentation readouts.

The antitumoral effect of nano-encapsulated tarin becomes more relevant when contextualized with previous evidence found by our group, in which tarin exhibited systemic immunomodulatory action. In cyclophosphamide-induced immunosuppression murine models, for example, tarin reduced leukopenia and genotoxicity, accelerated peripheral leukocyte recovery, and protected bone marrow cells from cytotoxic damage, all in a dose-dependent manner [23,92]. Thus, if confirmed, *HLA* modulation in cancer cells associated with reported cytoprotective effects could reinforce the hypothesis that tarin can act on both local and systemic immune mechanisms, representing a promising axis for future investigations.

### 3.3. Exclusive Proteins Identified at Different Nano-Encapsulated Tarin Treatment Times

The proteomic analysis revealed unique proteins at different time points, reinforcing the dynamic and progressive nature of the cellular response to nano-encapsulated tarin. Twenty-five exclusive proteins were identified after 24 h (absent both in the control and at 48 h) and 31 exclusive proteins at 48 h (Table S3). Among those present at 24 h, translation enzymes such as Lysyl-tRNA synthetase (*KARS*), Lysine-tRNA ligase, transcriptional zinc finger family regulators, immune response components (*HLA-DRB1*, immunoglobulin chains), and signaling modulators like MAP kinase 15 and Rho GTPase-activating protein 39 are noteworthy. This expressed set of proteins suggests early responses to nano-encapsulated tarin. Studies have demonstrated that *KARS* interacts with the laminin receptor (67LR) to promote migration and adhesion in tumor cells [93]. In 3D models, KARS also stimulates the spread of spheroids and modulates the microenvironment (i.e., M2 macrophage induction), favoring migration and invasion [94], and its inhibition reduces cell migration and proliferation in hepatocarcinoma cells [95]. Thus, the exclusive detection of *KARS* at 24 h and its absence at 48 h is consistent with the hypothesis that nano-encapsulated tarin interferes with the KARS-dependent migration pathway, and may contribute to decreased cell migration, as reported in our previous study [25].

An exclusive set of upregulated proteins, including class I major histocompatibility complex (*MHC I*) antigens, fibroblast growth factor (FGF), the ADAM-TS3 metalloproteinase, and proteins involved in DNA replication and repair such as *MCM7*, as well as cytoskeleton and extracellular matrix regulators, was observed after 48 h of nano-encapsulated tarin treatment. The presence of *MHC I* suggests an attempt to reestablish immune surveillance [96]. FGF, in turn, is involved in tumor adaptation processes, including resistance to conventional therapies [97]. *ADAM-TS3*, a metalloproteinase, plays a tumor suppressor role by restricting cell invasion, and its loss is associated with increased tumor aggressiveness [98]. *MCM7*, an essential DNA replication machinery component, is implicated in tumor progression and resistance to therapies, and its inhibition enhances chemotherapy efficacy [99]. These data suggest an early adaptive response of tumor cells to the stress induced by nano-encapsulated tarin that aims to repair damage, reorganize the extracellular matrix, maintain DNA integrity, and preserve cell viability. The initial stage is followed by the culmination of cell death, evidence of the modulation of the multifactorial effects of nano-encapsulated tarin in a tumoral environment.

The present study offers an extensive understanding of the quantification of differentially expressed proteins following the incubation of MDA-MB-231 cells with antitumoral nano-encapsulated tarin. A limitation of this study is the lack of experimental validation of key candidates (e.g., *CHEK1*, *PARK7*, *OPA1*, *CASP8*, *LAMA4*) by independent techniques, such as Western blotting or targeted proteomics. Based on this, future studies should focus on validating these proteins in additional TNBC models and correlate the response with distinct doses of nano-encapsulated tarin. On the other hand, as stated throughout the manuscript, even before these validations, the consistency of our results can be corroborated with existing studies, including our previous publications, where the same lineage and diversified techniques were employed to analyze the antitumoral mechanisms triggered by nano-encapsulated tarin before cell death [25]. In this previous study, we showed that the mechanisms underlying nano-encapsulated tarin-induced MDA-MB-231 cell death involve both apoptotic and autophagic pathways, with activation of caspase-3/7, cell cycle arrest at G0/G1, and the formation of acidic vacuolar organelles, which are characteristic of autophagosomes.

## 4. Conclusions

Nano-encapsulated tarin triggers a dynamic and progressive cellular response in breast adenocarcinoma cells (MDA-MB-231 lineage), indicating the activation of adaptive oxidative stress mechanisms and mitochondrial regulation, and cell cycle control takes place during the first 24 h, as suggested by the modulation of *PARK7/DJ-1*, *OPA1*, and *CHEK1*, unique proteins associated with translation, transcriptional regulation, and tumor remodeling. With continued exposure, these adaptative responses become insufficient, as demonstrated by the progression to cell death activation, and are marked by an increase in CASP8 expression after 48 h and suppression of proteins that control proliferation, migration, DNA repair, and structural organization (*CDK12*, *KIF21A*, *LAMA4*). The exclusive and transient detection of KARS at 24 h, absent at 48 h, also suggests inhibition of the *KARS–67LR* axis, which is associated with tumor migration and invasion. The set of unique proteins expressed at 48 h (*MHC I*, *FGF*, *ADAM-TS3*, *MCM7*) indicates a late repair attempt, but it is insufficient to prevent cell death. Thus, the proteomic analysis findings reinforce that nano-encapsulated tarin, a food-plant protein, could be a promising candidate for antitumor therapies that can modulate critical tumor survival and adaptation pathways, stimulating further investigations, including organoid model studies, in vivo assays, and the exploration of the immunomodulatory effects previously described for free tarin.

## Data Availability

The original contributions presented in this study are included in the article/Supplementary Material. Further inquiries can be directed to the corresponding author.

## Supplementary Materials

The following supporting information can be downloaded at https://www.mdpi.com/article/10.3390/pharmaceutics18091123/s1, Figure S1: Two-dimensional reversed-phase chromatographic gradient programs used for peptide fractionation prior to LC-MS/MS analysis. (Panel A) First-dimension chromatographic separation and six-step peptide fractionation. Peptides were fractionated into six step gradients according to the acetonitrile (ACN) concentration. Each fractionation step lasted 9.5 min and consisted of 11, 14, 17, 20, 50, or 80% ACN, respectively, maintained from 1.0 to 4.0 min. Following each fractionation step, the peptides were loaded onto the second column. (Panel B) Second-dimension reversed-phase chromatographic gradient program used for peptide separation. The 75-min gradient began at 7% acetonitrile and increased linearly to 40%, followed by a rapid increase to 85% acetonitrile, which was maintained briefly before the column was re-equilibrated at 7% acetonitrile until the end of the run. The chromatographic separation was coupled online to a SYNAPT XS mass spectrometer operated in HDMSE mode. Table S1: Comprehensive proteomic dataset obtained from MDA-MB-231 cells under control conditions and after exposure to nano-encapsulated tarin for 24 h and 48 h. Protein identification and label-free quantification were performed by 2D-LC-HDMSE. The table includes protein accession numbers, descriptions, ANOVA and q-values, protein abundance values, fold changes, statistical analyses, and variation coefficients for all detected proteins. Table S2: Differentially expressed proteins identified in MDA-MB-231 cells exposed to nano-encapsulated tarin. Proteins significantly upregulated or downregulated were organized according to treatment comparisons (ET24h vs C24h; ET48h vs C48h and ET48h vs ET24h), including protein accession, protein name, gene name, and log2 fold change values. Table S3: Proteins exclusively identified at 24 h and 48 h after treatment with nano-encapsulated tarin in MDA-MB-231 cells. The table includes protein accession numbers, protein names, and corresponding Gene IDs for proteins uniquely detected at each experimental time point.
